# Redox imbalance in COVID-19 pathophysiology

**DOI:** 10.1016/j.redox.2022.102465

**Published:** 2022-09-11

**Authors:** Nairrita Majumder, Vishal Deepak, Sarah Hadique, Drake Aesoph, Murugesan Velayutham, Qing Ye, Md Habibul Hasan Mazumder, Sara E. Lewis, Vamsi Kodali, Anthony Roohollahi, Nancy Lan Guo, Gangqing Hu, Valery V. Khramtsov, Richard J. Johnson, Sijin Wen, Eric E. Kelley, Salik Hussain

**Affiliations:** a Department of Physiology and Pharmacology, School of Medicine, West Virginia University, Morgantown, WV, USA; b Department of Internal Medicine, Section of Pulmonary, Critical Care and Sleep Medicine, School of Medicine, West Virginia University, Morgantown, WV, USA; c WVU Cancer Institute, West Virginia University, Morgantown, WV, USA; d Lane Department of Computer Science & Electrical Engineering, West Virginia University, Morgantown, WV, USA; e Department of Occupational and Environmental Health Sciences, School of Public Health, West Virginia University, Morgantown, WV, USA; f Department of Microbiology, Immunology & Cell Biology, West Virginia University, Morgantown, WV, USA; g Department of Biochemistry and Molecular Medicine, School of Medicine, West Virginia University, Morgantown, WV, USA; h Department of Medicine, Division of Renal Diseases and Hypertension, University of Colorado, Anschutz Medical Campus, Aurora, CO, USA; i Department of Epidemiology and Biostatistics, West Virginia University, Morgantown, WV, USA

**Keywords:** SARS-CoV-2, COVID-19, EPR, Electron paramagnetic resonance, Redox imbalance, Uric acid, Transcriptomics

## Abstract

**Background:**

The pathophysiologic significance of redox imbalance is unquestionable as numerous reports and topic reviews indicate alterations in redox parameters during corona virus disease 2019 (COVID-19). However, a more comprehensive understanding of redox-related parameters in the context of COVID-19-mediated inflammation and pathophysiology is required.

**Methods:**

COVID-19 subjects (n = 64) and control subjects (n = 19) were enrolled, and blood was drawn within 72 h of diagnosis. Serum multiplex assays and peripheral blood mRNA sequencing was performed. Oxidant/free radical (electron paramagnetic resonance (EPR) spectroscopy, nitrite-nitrate assay) and antioxidant (ferrous reducing ability of serum assay and high-performance liquid chromatography) were performed. Multivariate analyses were performed to evaluate potential of indicated parameters to predict clinical outcome.

**Results:**

Significantly greater levels of multiple inflammatory and vascular markers were quantified in the subjects admitted to the ICU compared to non-ICU subjects. Gene set enrichment analyses indicated significant enhancement of oxidant related pathways and biochemical assays confirmed a significant increase in free radical production and uric acid reduction in COVID-19 subjects. Multivariate analyses confirmed a positive association between serum levels of VCAM-1, ICAM-1 and a negative association between the abundance of one electron oxidants (detected by ascorbate radical formation) and mortality in COVID subjects while IL-17c and TSLP levels predicted need for intensive care in COVID-19 subjects.

**Conclusion:**

Herein we demonstrate a significant redox imbalance during COVID-19 infection affirming the potential for manipulation of oxidative stress pathways as a new therapeutic strategy COVID-19. However, further work is requisite for detailed identification of oxidants (O_2_^•-^, H_2_O_2_ and/or circulating transition metals such as Fe or Cu) contributing to this imbalance to avoid the repetition of failures using non-specific antioxidant supplementation.

## Graphical abstract

**Figure:**
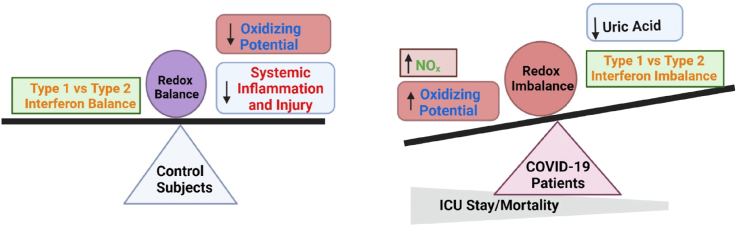

## 1 Introduction

Corona Virus Disease 2019 (COVID-19) is a form of sever acute respiratory and systemic illness caused by the Severe Acute Respiratory Syndrome Corona Virus 2 (SARS-CoV-2). During the last two decades, SARS-COV-2 is third corona virus shown to cause severe acute respiratory distress syndrome (ARDS) in humans. Previously, SARS-CoV-1 and Middle Eastern Respiratory Syndrome (MERS-CoV) outbreaks were reported in 2002 and 2013, respectively. COVID-19 differs from SARS-CoV-1 and MERS in terms of the sustained infectivity (human to human transmission) [[1], [2], [3]]. Since first confirmed case in December 2019, this disease has become the leading cause of morbidity and mortality throughout the world. According to the World Health Organization (WHO), 50.4 million confirmed cases and 6.2 million deaths related to COVID-19 have been reported as of April 20, 2022 [4]. The advent of vaccination and coordinated efforts for mass vaccination has resulted in a significant decrease in hospitalizations and mortality in the vaccinated individuals. However, the rapidly changing viral genetic landscape aided by a significant number of unvaccinated individuals is resulting in emergence of different variants with altered infection and pathogenic capacity. Given that vaccine hesitancy is an unfortunate reality and thus attainment of mass immunity may be very difficult; it is imperative to more clearly understand the pathophysiologic mechanism induced by the virus early during infection. This can help to identify better predictors of disease severity and outcome as well as will facilitate therapeutic development to control infection.

Oxidant stress is known to play important role in viral infections [[5], [6], [7], [8], [9]]. A defective oxidant balance can be deleterious and may result in sustained viral pathology [10]. Sustained viral replication requires a pro-oxidant environment, a milieu that also potentiates cytopathic effects [2,8,11]. The pathophysiologic roles of oxidant species in viral infections span from viral killing to aiding in the progression of infection through enhanced/uncontrolled inflammatory signaling and tissue injury [7,[12], [13], [14]]. Moreover, viral infections can induce sustained organelle stress responses (endoplasmic reticulum and mitochondria) and cell death mechanisms (apoptosis and autophagy) which are known to be controlled by redox status [[15], [16], [17], [18]]. Since the beginning of COVID-19, multiple reports have proposed a potential role of oxidant stress in its pathogenesis including the cytokine storm, endothelial cell death, coagulopathy and cellular hypoxia [6,[19], [20], [21], [22]]. However, only a limited number of original research studies explored these responses [[23], [24], [25], [26], [27]]. Of note, there are studies describing the role of gasotransmitters (hydrogen sulfide and nitric oxide), cellular glutathione and lipid peroxidation in the pathogenesis of COVID-19 [10,27,28]. Not surprisingly, another study confirmed impaired metabolism and redox function of cellular glutathione in SARS-CoV2 infection [29]. Similarly, structural damage to human serum albumin protein (considered contributory to the circulating antioxidant capacity) by COVID-19 induced oxidative stress was reported [26].

Multiple avenues for modulating the redox status for control of SARS-COV-2 and/or reduction in cellular damage such as improving cellular redox status by supplementation with glutathione, inhaled NO and ascorbic acid supplementation have been proposed to be potential therapeutic strategies and were components of clinical trials [[30], [31], [32]]. However, these approaches of non-specific augmentation of cellular antioxidant defense did not demonstrate durable salutary outcomes which was not surprising given similar findings in critically ill subjects with sepsis and ARDS (reviewed in detailed in Ref. [33]). A possible cause for the absence of positive outcomes in these trials is interference with normal immune response which further affirms the critical need to identify the exact nature of oxidant imbalance in COVID-19 pathogenesis to generate a more informed approach.

In order to develop effective therapeutic interventions, a crucial need exists to understand the host susceptibility factors driving the severity of COVID-19 infection. In this study, a cohort of hospitalized COVID-19 subjects and controls were evaluated during the first 72 h of admission after a positive PCR test. The oxidative parameters studied include: systemic oxidant/free radical levels, antioxidant capacity of serum as well as circulating uric acid levels. Furthermore, a transcriptomic analysis (mRNA sequencing) and real time PCR based gene expression analyses were performed on peripheral blood cells to identify unique as well as enriched gene sets in the subjects under intensive care and on the hospital floor. We further evaluated these parameters as the predictors of disease severity, duration of hospitalization, requirement of intensive care and mortality.

## 2 Methods

### 2.1 Study design and protocol

This is a prospective single center study conducted in an academic quaternary medical center of patients admitted with COVID-19 infection and acute hypoxemic respiratory failure. Institutional review board approval was obtained (IRB#2006030032A003). Electronic medical record (EMR) was reviewed to identify consecutive adult hypoxic patients admitted within the first 72 h with a confirmed COVID-19 reverse transcriptase-polymerase chain reaction (RT-PCR), after which the investigators obtained informed consent from the patient or their surrogate (in person or via telephone). Only one set of blood sample was collected per patient within 72 h of admission, the sample was then de-identified and taken to the basic science lab for analyses. Once the patients were enrolled, they were followed during the hospitalization and 30 days after discharge. Control subject samples were age, sex, ethnicity matched and obtained from the observational cohort called “Personalized Environment and Gene Study” (PEGS) formerly Environmental Polymorphism Registry (NCT00341237), at the National Institute of Environmental Health Sciences (NIEHS). These samples were collected before the appearance of first case of Covid-19.

### 2.2 Population and data collection

Inclusion criteria were hospitalized adult patients (≥18 years old) with hypoxic respiratory failure and positive COVID-19 RT-PCR within 72 h of the admission. Hypoxic respiratory failure was defined as arterial saturation ≤88% requiring supplemental oxygen. Exclusion criteria were age <18 year, pregnancy, incarceration, incidental finding of COVID-19 without concomitant hypoxia, or inability to obtain informed consent from the patient or healthcare surrogate within the first 72 h of admission. Classification criterion for categorizing COVID-19 patients was based on clinical picture as mild, moderate/severe and critical. Baseline demographics, comorbid conditions, measures of illness, Charlson Comorbidity Index (CCI) and hypoxia were collected. Severity of illness was measured as Sequential Organ Failure Assessment (SOFA) score, P/F ratio, use of high flow nasal cannula (HFNC), use of non-invasive positive pressure ventilation (NIPPV) and invasive mechanical ventilation, or requirement of renal replacement therapy. Data was aggregated using the HIPAA-compliant Research Electronic Data Capture (REDCap) electronic data capture tool.

### 2.3 RNA-sequencing data generation

NextSeq2000 was used to generate the sequencing data of 36 million clusters (or 72 million F + R reads) per sample with the P3 flow cell from globen depleted samples at Marshal University Genomics Core (Huntington, WV, USA). RNA-Seq data analysis follows our previous work [34,35]: briefly, RNA-Seq read aligned to human genome by subread [36], read counts summarized based on RefSeq gene annotation by featurecount [37], expression level quantification by RPKM [38] with an in-house script, visualization of gene expression by MeV [39], prediction of differentially expressed (DE) genes by EdgeR (FDR = 0.05; |log2FC| > 1, and log2(Count Per million) > 0), and gene set enrichment analysis against hallmark gene sets by GSEA [40,41].

#### 2.3.1 Functional enrichment analysis using ToppGene

ToppFun application of the ToppGene suite [42] was used to find functional enrichment of molecular pathways and diseases. The genes that were significantly differentially expressed were selected as inputs to ToppFun. The significantly differentially expressed gene was defined as the gene with a log2 CPM higher than 0, a log2 fold change higher than 2 or lower than −2, and a false discovery rate (FDR) lower than 5%. Retrieval of Pathway Information from MSigDB Database GSEA MSigDB [43] v7.4 (www.broad.mit.edu/gsea/msigdb/accessed on November 23, 2021) pathway information was used in this study. This database was used to retrieve the pathways that were relevant to specific processes including albumin, glutathione, oxidative stress, phagocytosis, and reactive oxygen species.

### 2.4 Electron paramagnetic resonance (EPR) spectroscopy

EPR spectrometry was used to measure the oxidizing potential of human serum samples using the spin probe 1-hydroxy-3-carboxymethyl-2,2,5,5-tetramethyl-pyrrolidine (CMH) (Enzo Life Science) as described previously [44,45]. Briefly, 0.1 ml of serum was incubated with the EPR spin probe CMH (0.2 mM) or sodium ascorbate (1 mM) for 30 min at 37 °C. CMH (EPR inactive) and sodium ascorbate (Sigma Aldrich) are oxidized by reactive species in the serum to 3-carboxymethyl-2,2,5,5- tetramethyl-pyrrolidinyloxy radical (CM^•^; EPR active) and ascorbate radical (EPR active). After incubation, samples were flash frozen in liquid nitrogen and stored at −80 °C until analysis. At the time of EPR measurements, frozen samples were thawed to room temperature and loaded (50 μL) into glass capillary tubes that were sealed on one end using Critoseal clay and placed inside the 4 mm (O.D.) EPR quartz tube. The quartz tube was positioned inside the resonator/cavity and spectra recorded at room temperature.

EPR spectra were recorded using a Bruker EMXnano spectrometer (Bruker BioSciences, Billerica, MA, USA) operating at X-band with a 100 kHz modulation frequency. The following EPR instrument settings were used: microwave frequency, 9.617 GHz; sweep width, 100 G (20 G for ascorbate); microwave power, 20 mW; modulation amplitude, 1 G (0.5 G for ascorbate); modulation frequency, 100 kHz; receiver gain, 50 dB (60 dB for ascorbate); time constant, 10.24 ms (2.56 ms for ascorbate); conversion time, 30 ms (10 ms for ascorbate), sweep time, 30 s (10 s for ascorbate); number of scans, 1 (30 for ascorbate).

Data acquisition was performed using Bruker Xenon_nano software. Data processing was performed using GraphPad Prism 8 software.

### 2.5 Nitrite + nitrate (NO_x_) determination

Total plasma nitrite (NO_2_^−^) + nitrate (NO_3_-) was determined using enhanced chemiluminescence (Sievers, NOA). Briefly, plasma aliquots (20 μL) were injected directly into the reaction chamber where nitrate and nitrite are reduced to nitric oxide using vanadium (III) chloride in hydrochloric acid at 90 °C. Each patient's plasma level of NO_x_ was determined using the mean values of 3 separate injections (20 μL). Standard curves for quantification were performed using NaNO_3_.

### 2.6 Serum antioxidant assay

Ferric reducing ability of serum (FRAS) assay was used to determine the alteration in total antioxidants in serum from patients. This is an adaption of the Ferric reducing ability of plasma (FRAP) assay by Benzie et al. [46]. The assay relies on development of intense blue color (measured by absorption at 586 nm) formed due to reduction of ferric-tripyridyltriazine (Fe^3^-TPTZ) to its ferrous (Fe^2^) form depending on the presence of a reductant (antioxidants in the serum).

$$Fe^{3+}-TPTZ+Reducing\;Antioxidant\;\to \;Fe^{2+}-TPTZ\;\left(\mathrm{Intense}\;\mathrm{Blue}\;\mathrm{at}\;595\;\mathrm{nm}\right)$$

To quantify the level of antioxidants present, 12.5 μL of human serum thawed on ice was reacted with 250 μL of a volume mixture consisting of 0.2021 g of sodium acetic trihydrate and 1.060 ml of glacial acetic acid (Alfa Aesar, Haverhill, MA; Cat # 36289) in 100 ml of deionized water, 0.0946 g of TPTZ (2,4,6-tri (2-pyridyl)-*s*-triazine)(Sigma-Aldrich, Cat #T1253) and 1.2 ml of 1 M HCl in 30 ml of deionized water and 0.1635 g of FeCl3·6H_2_O (Sigma-Aldrich, Cat # 44944) in 30 ml of deionized water respectively. A commercial human serum (Sigma-Aldrich, St. Louis, MO; Cat #P2918) was used as a control to compare relative change in the antioxidant levels. After 10 min of reaction at room temperature, the antioxidant levels were monitored by measuring absorbance at 586 nm. Experiment was performed in a 96 well plate. The human serum samples were thawed on ice and randomly divided into three groups. Three technical replicates were performed on each human serum sample. Background turbidity was normalized by subtracting the absorbance at 680 nm. The control commercial human serum was included on three plates and the data from each individual serum sample was normalized and presented as % change in antioxidants compared to this control serum. Each data point is an average of the three technical replicates.

### 2.7 Uric acid assay

Uric acid levels in plasma were determined using reverse-phase HPLC coupled to an electrochemical detector as we previously described [47].

### 2.8 Mesoscale Discovery multiplex 54-plex assay

The immune/inflammatory mediator/injury marker concentrations in the serum samples were analyzed using the Mesoscale Discovery V-PLEX ELISA Assay (MSD 54-plex) (Meso Scale Diagnostics, Rockville, MD). Briefly, samples and controls were diluted (as per manufacturer's protocol) with Diluents, followed by incubation in 96 well plate for 2 h at room temperature with constant shaking at 500 rpm. Plates were washed 3 times with at least 150μL/well wash buffer, followed by addition of 25 μL of Detection Antibody per well, and incubation at room temperature with shaking for 2 h. The plates were washed 3 times with MSD Wash Buffer. The plates were read and analyzed in MSD QuickPlex SQ120 immediately after addition of 150 μL/well of 2X Read Buffer. The concentrations were calculated as “calculated concentration means” on the MSD Discovery Workbench 4.0 software. All standards were run in duplicates.

### 2.9 Real-time RT PCR

RNA from blood was extracted using Qiagen QIAamp RNA Blood Mini Kit (Qiagen, Germantown, MD) as per manufacturer's instructions. RNA concentration was measured using Nanodrop. Reverse Transcription was performed using the Applied Biosystems™ High-Capacity cDNA Reverse Transcription Kit (Thermo Fisher Scientific, Waltham, MA) as per manufacturer's instruction. PCR reaction was performed in duplicate using AriaMx Real time PCR Machine (Agilent, Santa Clara, CA) applying a Syber Green (Thermo Fisher Scientific, Waltham, MA) chemistry. Primer sequences details can be found in Supplemental File S1-Table 1. The relative expression level of genes was measured as fold change to control subjects using the comparative threshold method with 18S as internal control. Data was analyzed using ΔΔCt method, where fold change = 2^−ΔΔCt^.

### 2.10 Statistical analysis

All data analysis was performed using the statistical software R, version 3.6.3. Mean and standard deviations were reported for continuous variables, and proportions for categorical variables. Differences between groups were assessed by the Wilcoxon rank test for continuous variables and Chi-square test or Fisher exact test as appropriate for categorical variables. Pearson's correlation or Spearman's correlation was estimated in the correlative analysis between two continuous variables. Protocol labs were analyzed as continuous variables and also by stratifying them as dichotomous (normal vs. abnormal). Linear regression model and logistic regression model were used in the multivariate analysis on the continuous variables or the dichotomized outcome variables respectively. Kaplan Meier method and log-rank test were used to assess survival functions and 30-day mortality difference between the two groups. All statistical tests were 2-sided, and an alpha value of 0.05 was used to determine statistical significance. Power analysis was performed based on statistical software PASS version 15.0.5. In the correlative analysis on 30-day mortality, our sample size achieves 80% power to detect an effect size of 0.90 on a biomarker between death (n = 14) and alive (n = 37) using a two-sample 2-sided t-test at a significance level of 0.05. Meanwhile, this sample size achieves 80% power to detect a Pearson or Spearman correlation between 0.0 (null) and 0.35 (alternative) using a two-sided test at a significance level of 0.05.

## 3 Results

### 3.1 Patient outcomes

Sixty-four patients, and 32 controls were enrolled in the study. Out of 32 controls, detailed demographic data was available for 19 subjects, which was used for comparison (Table 1). The majority of the patients were male (n = 53, 63.9%), Caucasian (n = 78, 94%) and above 60 years of age (n = 50, 60.2%). 50.6% (n = 42) subjects were either former or active smokers. Except smoking status, there was no statistically significant difference between patients and controls. Among the patients, mean BMI was 33.31 ± 10.24 reflecting an obese population and most common co-morbidities were hypertension (n = 51, 87.9%), hyperlipidemia (n = 34, 58.6%) and diabetes mellitus type II (n = 32, 55.5%) with mean CCI 4.5 ± 3.06. Most common presentations to the hospital included shortness of breath (76.3%), hypoxia (62.7%) and fever (44.1%) (Supplementary File S1-Table 2). Forty-two (68.9%) patients were clinically classified to have moderate to severe disease due to COVID-19 infection (Table 2). Regarding severity of illness, mean SOFA score and P/F ratio were 3.66 ± 3.17 and 268.93 ± 140.68 respectively. Fourteen (21.9%) patients required transfer to intensive care unit, whereas 18.6% (n = 11) required intubation and invasive mechanical ventilation (IMV). Seven (11.5%) patients required hemodialysis. Venous thromboembolism was not prevalent in our study patients with only 2 (3.3%) patients was found to have either DVT or PE. Mean length of stay in ICU and hospital in days was 7.50 ± 3.52 and 7.20 ± 5.84. Fifteen (23.4%) patients died within 30 days of the enrollment into the study. 30 days mortality was associated with the history of smoking, organ dysfunction, hemodynamic instability, and respiratory distress on presentation (p-value <0.05) (Table 3). Furthermore, NIV-PPV, IPPV and hemodialysis during hospital admission were associated with increased mortality within 30 days. (p-value < 0.05).

**Table 1:** Cohort characteristics. BMI=Body Mass Index; CAD=Coronary artery disease; CCI=Charlson Comorbidity Index; CHF=Congestive heart failure; CKD=Chronic kidney disease; COPD=Chronic obstructive pulmonary disease.

Variables Mean ± SD or n (%)	All Subjects with or without Serum data				Subjects with Serum data			
	Controls	Patients	Total	Chi-square test	Controls	Patients	Total	Chi-square test
	(n = 19)	(n = 64)	(N = 83)	p-value	(n = 19)	(n = 53)	(N = 72)	p-value
Gender n (%)								
Female	5 (26.3)	25 (39.1)	30 (36.1)	0.457	5 (26.3)	18 (34)	23 (31.9)	0.744
Male	14 (73.7)	39 (60.9)	53 (63.9)		14 (73.7)	35 (66)	49 (68.1)	
Age								
<60	10 (52.6)	23 (35.9)	33 (39.8)	0.299	10 (52.6)	19 (35.8)	29 (40.3)	0.314
≥60	9 (47.4)	41 (64.1)	50 (60.2)		9 (47.4)	34 (64.2)	43 (59.7)	
Race								
Caucasian	18 (94.7)	60 (93.8)	78 (94)	0.677	18 (94.7)	49 (92.5)	67 (93.1)	0.671
African-American	1 (5.3)	2 (3.1)	3 (3.6)		1 (5.3)	2 (3.8)	3 (4.2)	
Unknown	0 (0)	2 (3.1)	2 (2.4)		0 (0)	2 (3.8)	2 (2.8)	
Smoking								
Never Smoker	6 (31.6)	26 (40.6)	32 (38.6)	0.001	6 (31.6)	24 (45.3)	30 (41.7)	0.002
Former Smoker	3 (15.8)	31 (48.4)	34 (41)		3 (15.8)	23 (43.4)	26 (36.1)	
Current Smoker	4 (21.1)	4 (6.2)	8 (9.6)		4 (21.1)	3 (5.7)	7 (9.7)	
N/A	6 (31.6)	3 (4.7)	9 (10.8)		6 (31.6)	3 (5.7)	9 (12.5)	
Hypercholesteremia								
No	9 (50)	30 (46.9)	39 (47.6)	0.974	9 (50)	25 (47.2)	34 (47.9)	0.948
Yes	9 (50)	34 (53.1)	43 (52.4)		9 (50)	28 (52.8)	37 (52.1)	
CAD								
No	11 (91.7)	39 (60.9)	50 (65.8)	0.084	11 (91.7)	35 (66)	46 (70.8)	0.158
Yes	1 (8.3)	25 (39.1)	26 (34.2)		1 (8.3)	18 (34)	19 (29.2)	
Asthma								
No	15 (78.9)	56 (87.5)	71 (85.5)	0.576	15 (78.9)	47 (88.7)	62 (86.1)	0.506
Yes	4 (21.1)	8 (12.5)	12 (14.5)		4 (21.1)	6 (11.3)	10 (13.9)	
Cancer								
No	17 (89.5)	55 (85.9)	72 (86.7)	0.989	17 (89.5)	45 (84.9)	62 (86.1)	0.914
Yes	2 (10.5)	9 (14.1)	11 (13.3)		2 (10.5)	8 (15.1)	10 (13.9)

**Table 2:** Covid-19 patient cohort characteristics. SOFA=Sequential organ failure assessment; P/F ratio = arterial pO2 divided by the fraction of inspired oxygen; HFNC= High flow nasal cannula; NIV-PPV = noninvasive positive pressure ventilation; IPPV = invasive positive pressure ventilation; MICU = Medical Intensive Care Unit; VTE=Venous thromboembolism; DVT = Deep venous thrombosis; PE=Pulmonary embolism; ACS = Acute coronary syndrome.

COVID-19 category	
Mild	7 (11.5%)
Moderate/severe	42 (68.9%)
Critical	12 (19.7%)
Severity of illness	
SOFA	3.66 + 3.17
P/F ratio	268.93 + 140.68
Use of HFNC, %	14 (23.7%)
Use of NIV-PPV, %	9 (15.3%)
Transfer to MICU	14 (21.9%)
Use of IPPV	11 (18.6%)
Hemodialysis	7 (11.5%)
Outcomes	
Presence of VTE	2 (3.3%)
DVT	1 (50%)
PE	1 (50%)
Arterial thrombus	0
Stroke	0
ACS	4 (6.6%)
ICU length of stay (Days)	7.50 + 3.52
Hospital length of stay (Days)	7.20 + 5.84

**Table 3:** Correlation of 30-day mortality and intensive care unit admission with clinical parameters.

Variables Mean ± SD or n (%)	n	ICU Care		Fisher test	30day-mortality		Fisher test
Smoking		No	Yes	p-value	No	Yes	p-value
Never Smoker	26 (40.6)	23 (88)	3 (12)	0.077	23 (88)	3 (12)	0.05
Former Smoker	31 (48.4)	22 (71)	9 (29)		21 (68)	10 (32)	
Current Smoker	4 (6.2)	4 (100)	0 (0)		4 (100)	0 (0)	
Not available	3 (4.7)	1 (33)	2 (67)		1 (33)	2 (67)	
Organ dysfunction							
No	58 (90.6)	46 (79)	12 (21)	0.604	47 (81)	11 (19)	0.023
Yes	6 (9.4)	4 (67)	2 (33)		2 (33)	4 (67)	
Hemodynamic instability							
No	61 (95.3)	50 (82)	11 (18)	0.009	49 (80)	12 (20)	0.011
Yes	3 (4.7)	0 (0)	3 (100)		0 (0)	3 (100)	
Respiratory distress							
No	54 (84.4)	49 (91)	5 (9)	<0.001	49 (91)	5 (9)	<0.001
Yes	10 (15.6)	1 (10)	9 (90)		0 (0)	10 (100)	
CKD							
No	48 (75)	42 (88)	6 (12)	0.004	38 (79)	10 (21)	0.498
Yes	16 (25)	8 (50)	8 (50)		11 (69)	5 (31)	
COVID severity							
Mild	7 (11.5)	6 (86)	1 (14)	<0.001	7 (100)	0 (0)	<0.001
Moderate	42 (68.9)	41 (98)	1 (2)		38 (90)	4 (10)	
Severe	12 (19.7)	0 (0)	12 (100)		1 (8)	11 (92)	
NIV-PPV							
No	55 (85.9)	43 (78)	12 (22)	1	45 (82)	10 (18)	0.027
Yes	9 (14.1)	7 (78)	2 (22)		4 (44)	5 (56)	
IPPV							
No	53 (82.8)	50 (94)	3 (6)	<0.001	49 (92)	4 (8)	<0.001
Yes	11 (17.2)	0 (0)	11 (100)		0 (0)	11 (100)	
Hemodialysis							
No	54 (88.5)	45 (83)	9 (17)	0.005	43 (80)	11 (20)	0.055
Yes	7 (11.5)	2 (29)	5 (71)		3 (43)	4 (57)	
30-day mortality							
No	49 (76.6)	46 (94)	3 (6)	<0.001	–	–	–
Yes	15 (23.4)	4 (27)	11 (73)		–	–	–

#### 3.1.1 Systemic inflammation and injury markers

**Fig. 1:**
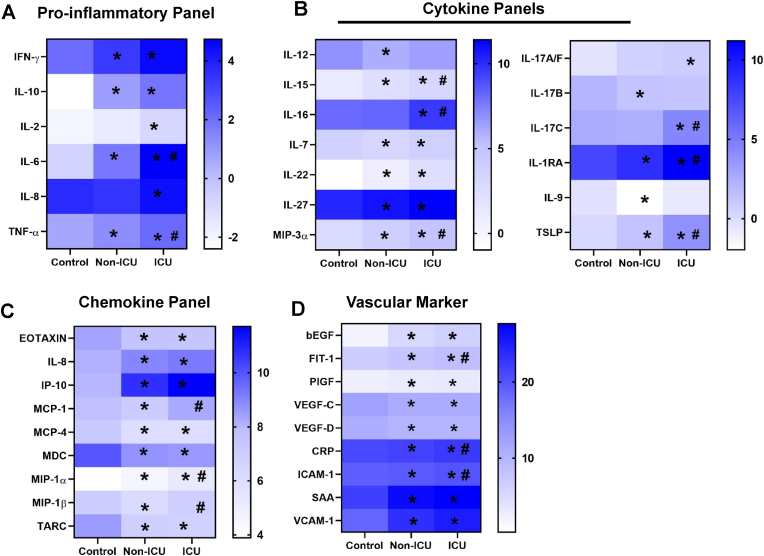
Multiplex analyses of serum samples. Heat map representing A) Pro-inflammatory panel B) Cytokine panels C) Chemokine panel D) Vascular panel marker quantification in serum samples from Control subjects (n = 19), non-ICU (n = 38) and ICU (n = 13) admissions. The values are presented as Log2 transforms. Data analyzed by Kruskal-Wallis test followed by Dunn's multiple comparison test. *p < 0.05 vs control, #p < 0.05 ICU vs non-ICU patients.

We performed multiplex assays on serum samples to quantify a variety of systemic inflammatory markers including pro-inflammatory cytokines, chemokines, growth factors, and vascular pathology mediators (Fig. 1). We observed a significant increase in the serum levels of diverse markers of inflammation and vascular injury. Significant differences in the levels of IL-6, TNF-α, IL-15, IL-16, MIP-3α, MIP-1ꞵ, IL-1rA, TSLP, MIP-1α, FIT1, CRP and ICAM-1 were quantified in subjects admitted to ICU compared with subjects on hospital floor. Moreover, significant changes in multiple IL-17s (IL-17A/F, IL-17B, IL-17C) and IL-10 family cytokines (IL-10, IL-22) were also noted. In general, these findings confirmed significantly greater immune modulation in subjects admitted to ICU compared with hospital floor (non-ICU) subjects. Among the vascular injury and angiogenesis markers, patients admitted to ICU had a significant increase in CRP, ICAM-1, and Flt-1 compared to the patients admitted to the hospital. Several mediators such as Tie-2, VEGFA, IL-17D, IL-3, GM-CSF, IL-1α, IL-5, Eotaxin 3, IL-12p70 and IL-13 were not significantly different between COVID-19 and control subjects (data not shown). Next, we evaluated the correlation of 30-day mortality and length of hospitalization with the serum levels of these mediators (Table 4, Table 5). We observed a significant association of 30-day mortality with 21 mediators (Table 4). Spearman correlation analyses demonstrated a significant (p ≤ 0.5) positive association of 15 serum markers, while 5 serum markers were negatively associated with length of hospitalization (Table 5).

**Table 4:** Association of circulating soluble factors with 30-day mortality.

	30-day Mortality
	Wilcoxon rank test p-value
CRP	0
ICAM-1	0.001
SAA	0.002
VCAM-1	0
ꞵFGF	0.078
FIT-1	0.002
IL-17c	0.001
IL-1Rα	0.016
TSLP	0
IL-22	0.068
MIP-3α	0.022
IL-15	0
IL-16	0
Eotaxin	0.043
IP-10	0.048
MCP-1	0.004
MIP-1α	0.016
MIP-1ꞵ	0.011
IL-10	0.008
IL-6	0
TNF-α	0.017

**Table 5:** Spearman correlation of circulating soluble factors with length of hospitalization.

	Length of hospitalization
	Spearman correlation (p-value)
CRP	0.32 (0.010)
ICAM-1	0.08 (0.013)
SAA	0.17 (0.015)
VCAM-1	0.27 (0.002)
VEGF-C	−0.23 (0.042)
IL-17c	0.24 (0.037)
IL-1RA	0.17 (0.040)
TSLP	0.24 (0.002)
IL-22	0.33 (0.003)
IL-12	−0.11 (0.023)
IL-15	0.33 (0.0)
IL-16	0.16 (0.001)
IP-10	0.28 (0.011)
MCP-1	0.23 (0.002)
MCP-4	−0.14 (0.082)
MDC	−0.32 (0.002)
IFN.r	0.08 (0.016)
IL-10	0.15 (0.005)
IL-6	0.15 (0.001)

#### 3.1.2 Enriched pathways of significantly differentially expressed genes (mRNA-Sequencing)

**Fig. 2:**
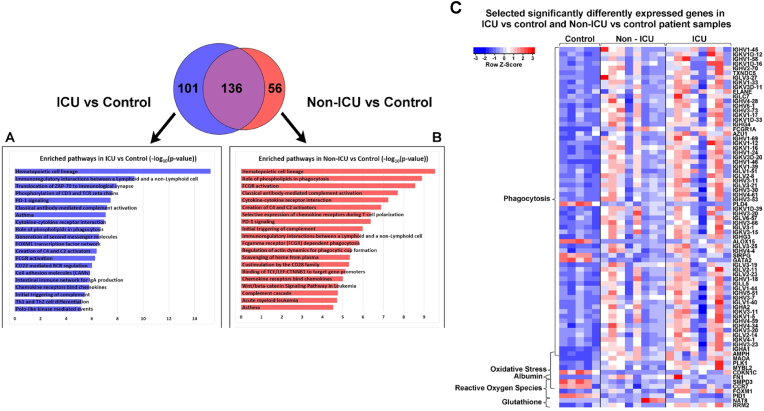
mRNA seq analyses. Significant pathways associated with differentially expressed genes in COVID-19 patient samples in the comparison of ICU vs Control and Non-ICU vs Control group. Venn diagram shows the number of significantly differentially expressed genes in ICU vs Control group, non-ICU vs Control group, and the common genes between these two groups. Bar charts show the top 20 significantly enriched pathways of significantly differentially expressed genes in ICU vs control (A) and non-ICU vs control (B) patient samples. ToppGene functional enrichment analysis was used. Detailed information is provided in supplementary file S1 sheet 3–6. C) Heatmap of the expression of the selected significantly differentially expressed genes in ICU vs control and non-ICU vs control patient samples. These genes are relevant to the following processes including albumin, glutathione, oxidative stress, phagocytosis, and reactive oxygen species. The RPKM values shown in the heatmap were normalized by each row (gene). Detailed information is provided in supplementary file S2 sheet 4.

In the circulating cells, 237 genes were significantly differentially expressed in ICU vs control group, 192 genes significantly differentially expressed in non-ICU vs control group, 136 genes common in both groups (log2 CPM >0, |log2 fold change| > 2, and FDR <5%). These 237 differentially expressed genes and 136 differentially expressed genes were used in ToppGene analysis to identify the enriched pathways and diseases for ICU vs control group and non-ICU vs control group, respectively. Fig. 2 A, B shows the top 20 significantly enriched pathways based on differently expressed genes in COVID-19 patients who were admitted to ICU and who were not admitted to ICU (non-ICU vs control) patient samples. Role of phospholipids in phagocytosis was ranked as a top dysregulated pathway in both COVID-19 patients in both ICU and non-ICU groups compared with control. Detailed information was provided in *Supplementary file S2*. The molecular pathways relevant to phagocytosis were further investigated and the expression of significantly differentially expressed genes in this molecular pathway is shown in Fig. 2C.

##### 3.1.2.1 Gene set enrichment analyses (GSEA)

**Fig. 3:**
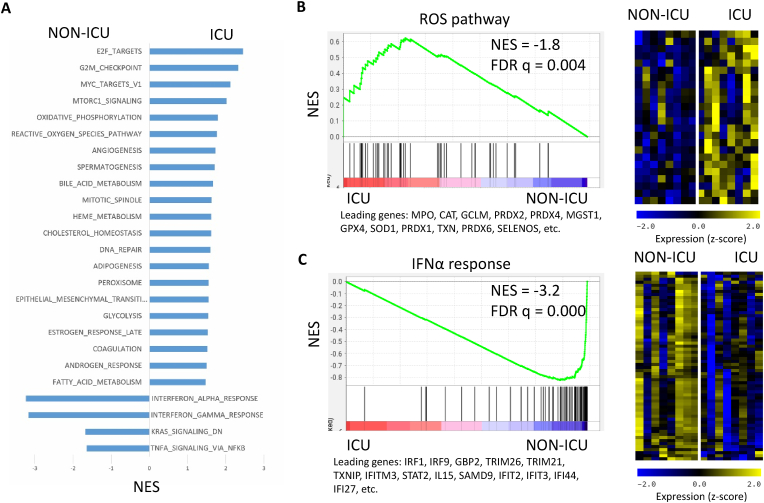
Gene set enrichment analyses. A) Bar plots for normalized enrichment scores from gene set enrichment analysis of the expression changes (ICU vs NON-ICU) against MSigDB Hall mark gene sets. Shown are FDR q < 0.05. B) Gene set enrichment analysis of the genes sorted by the expression changes (ICU vs NON-ICU; the blue-to-red spectrum) against gene set “ROS Pathway” (vertical bars) with top leading genes shown on the left bottom and heatmap visualization of the gene expression for all leading genes shown on the right. C) similar to B but for the gene set “IFN-α response). (For interpretation of the references to color in this figure legend, the reader is referred to the Web version of this article.)

GSEA was performed using MSIGDB Hallmarks and top enriched gene sets were compared in patients admitted to ICU to those that were admitted to hospital floor. Fig. 3A present top enriched gene sets in either subjects admitted to ICU or non-ICU subjects (Fig. 3A). More details on the selected pathways, gene sets and genes are provided in *Supplementary file S3*. Our laboratory has specific interest in redox related mechanisms, so among the top enriched gene sets in ICU subjects we further elaborated leading genes in “reactive oxygen species (ROS)” pathway (Fig. 3 B). Among the gene sets that were most significantly enriched in non-ICU subjects, “interferon-α response” pathway is detailed in Fig. 3C.

#### 3.1.3 Real-time PCR validation of interferon-alpha expression changes

**Fig. 4:**
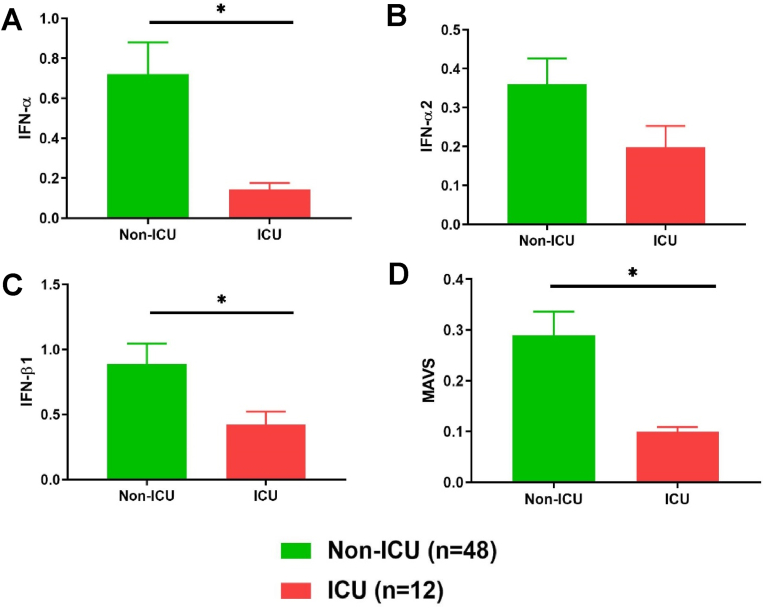
Real-time PCR gene expression Analyses. Expression levels of A) IFN- α, B) IFN-α2, C) IFN-β1, and D) MAVS in the buffy coats from control subjects and COVID-19 subjects.) Data represents comparison between non-ICU (n = 51) and ICU (n = 13) admissions. Data is normalized to fold change to control and analyzed by non-parametric *t*-test. Data is analyzed by Mann Whitney test. *p < 0.05, ***p < 0.001, ****p < 0.0001.

Next, we evaluated mRNA expression for some interferons (*IFN-α*, *IFN-α2*, *IFN-ꞵ1*) and a related gene (*MAVS*) (Fig. 4). We observed significantly lower expression of *IFN-α*, *IFN-ꞵ1* and *MAVS* in subjects admitted to ICU vs non-ICU subjects (Fig. 4 A-D).

#### 3.1.4 EPR spectroscopic studies of reactive oxidants in human serum samples

**Fig. 5:**
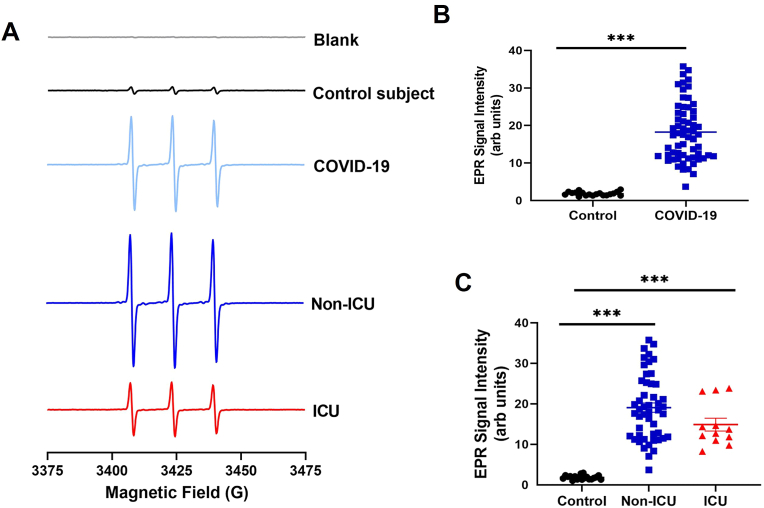
EPR studies of oxidation of CMH by human serum samples. A) Representative room temperature X-band EPR spectra of CM^•^ radical. (A) Blank (CMH without serum in PBS), Control subject (serum from non-COVID-19 subjects), COVID-19 (serum from COVID-19 subjects), Non-ICU, (serum from non-ICU COVID-19 subjects), and ICU (serum from ICU COVID-19 subjects). B) Plot of EPR signal intensity of CM^•^ radical in the serum samples from control (n = 37) and COVID-19 patients (n = 61). C) Plot of EPR signal intensity of CM^•^ radical in the serum samples from control subjects (n = 37) and COVID-19 subjects with non-ICU (n = 49) and ICU (n = 12) hospital admissions. Data analyzed by Mann Whitney test (B) or by Kruskal-Wallis test followed by Dunn's multiple comparison test(C). ***p < 0.001.

To assess the presence of oxidants in the serum, a nonspecific EPR technique was used that reacts with a broad range of one-electron oxidation partners such as O_2_^•^^-^, O=NOO^−^ and transition metals. EPR spectra of human serum samples incubated with the hydroxylamine spin probe CMH for 30 min at 37 °C are shown in Fig. 5. The EPR spectrum exhibits a characteristic triplet pattern due to contributions of the nitrogen atom with an isotropic hyperfine coupling constant of 16 G [44,45]. A very weak EPR signal was obtained from the control solution containing CMH in PBS without serum, as shown in Fig. 5A. Incubation of human serum (both ICU and non-ICU) and CMH gives strong EPR signals. Quantification of the first peak of the spectra is presented in Fig. 5B and C. Our studies demonstrated significantly greater signal intensity in COVID-19 subjects compared with control subjects (Fig. 5B). Significant differences were not observed between subjects admitted to ICU versus non-ICU subjects (Fig. 5 C).

##### 3.1.4.1 Ascorbate radical generation studies

**Fig. 6:**
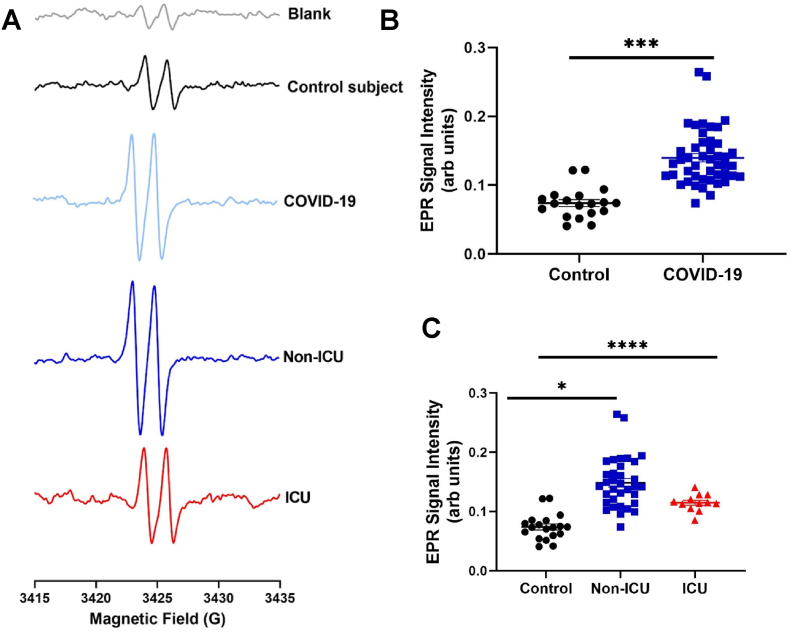
Ascorbate radical generation quantification by EPR. A) Representative room temperature X-band EPR spectra of ascorbate radical. Blank (ascorbate without serum in PBS), Control subject (serum from non-COVID-19 subjects), COVID-19 (serum from COVID-19 subjects), Non-ICU (serum from non-ICU COVID-19 subjects) and, ICU (serum from ICU COVID-19 subjects). B) Plot of EPR signal intensity of ascorbate radical comparing in the serum samples from control (n = 19) and COVID-19 patients (n = 61). C) Plot of EPR signal intensity of ascorbate radical in the serum samples from control subjects (n = 37) and COVID-19 subjects with Non-ICU (n = 49) and ICU (n = 12) hospital admissions. Data analyzed by Mann Whitney test (B) or by Kruskal-Wallis test followed by Dunn's multiple comparison test(C). *p < 0.05, ***p < 0.001, ****p < 0.0001.

Similarly, serum samples were incubated with ascorbate (exogenous) and EPR studies were carried out. The EPR spectra of ascorbate radical from the serum samples are shown in Fig. 6A. The ascorbate radical signal intensity from the COVID-19 patient serum samples is much greater than corresponding healthy subjects Fig. 6B. Similar to the CMH studies above, a significant difference between the levels of ascorbate radical in the subjects admitted to ICU and non-ICU patients was not observed. These results serve as a barometer to establish a greater level of oxidants/transition metals in the serum (Fig. 6C).

#### 3.1.5 Serum NO_x_ levels

**Fig. 7:**
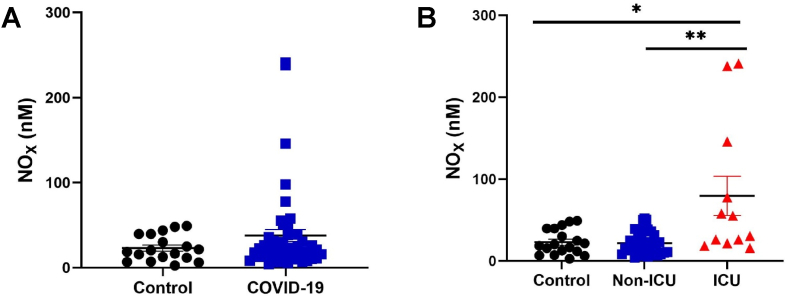
NOx estimation in the serum. NOx (Nitrite + Nitrate) levels in serum from control subjects (n = 18) and COVID-19 patients (n = 48). B) NOx levels comparing serum from control subjects (n = 18) with non-ICU (n = 36) and ICU (n = 12) admissions. Data is analyzed by Mann Whitney test (A) and Kruskal-Wallis test followed by Dunn's multiple comparison test (B). *p < 0.05, ***p < 0.001, ****p < 0.0001.

As a surrogate marker for nitric oxide abundance, levels of NO_x_ (nitrite + nitrate) were measured in serum from healthy and SARS-CoV-2 infected subjects (Fig. 7). A significant difference in serum NO_x_ levels between control subjects and SARS-CoV-2 infected subjects was not observed (Fig. 7A). However, significantly greater NO_x_ levels were noted in the critically ill subjects (subjects admitted to ICU) compared to the subjects to non-ICU subjects (admitted to hospital floor) (Fig. 7B).

#### 3.1.6 Changes in serum antioxidant levels

**Fig. 8:**
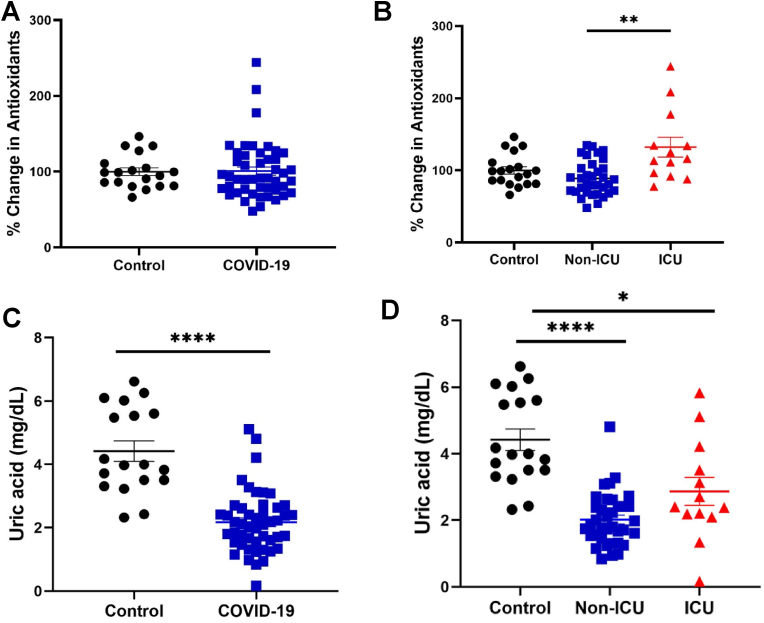
Antioxidant capacity and uric acid measurements. A) Serum antioxidant capacity measured by FRAS assay between control subjects (n = 19) and COVID-19 patients (n = 47). B) FRAS assay comparing serum from control subjects (n = 19) with non-ICU (n = 34) and ICU (n = 13) admissions. C) Uric acid concentration in serum from control subjects (n = 19) and COVID-19 patients (n = 48). B) Uric acid concentration comparing serum from control subjects (n = 19) with non-ICU (n = 35) and ICU (n = 13) admissions. Data is analyzed by Mann Whitney test (A, C) and Kruskal-Wallis test followed by Dunn's multiple comparison test (B and D). *p < 0.05, ***p < 0.001, ****p < 0.0001.

FRAS assay was performed to evaluate antioxidant capacity of the serum from control and patient samples (Fig. 8). Overall, no significant difference was observed in serum antioxidant capacity between healthy and COVID-19 subjects (Fig. 8A). Interestingly, an increase in antioxidant capacity was observed in subjects admitted to ICU compared to control subjects and the subjects admitted to hospital floor (non-ICU subjects) (Fig. 8B). However, uric acid levels in the serum were significantly decreased in COVID-19 subjects compared to healthy subjects (Fig. 8C). This change was evident in subjects admitted to ICU as well as subjects admitted to the hospital floor (non-ICU), however, no significant difference between the two groups was observed.

#### 3.1.7 Multivariate analyses for the biomarkers

Multivariate analyses were performed to examine potential biomarkers for 30- day mortality and ICU care using logistic regression models (Table 6). A significant association between 30-day mortality and serum levels of ICAM-1 (p = 0.035, OR 1.028, CI (1.002–1.055), VCAM-1 (p = 0.043, OR 2.154, CI (1.025–4.525) and ascorbate radical (p = 0.042, OR < 0.001, CI (<0.001–0.0124) was observed. Moreover, a significant association between ICU care and serum levels of TSLP (p = 0.031, OR 1.242, CI (1.020–1.511) and IL-17C (p = 0.050, OR 1.140, CI (1.000–1.300) was observed.

**Table 6:** Multivariate analyses for identification of early biomarkers of the outcomes (30-day mortality and ICU care).

Multivariate linear model adjusting for confounding variables											
	Value	S.E.			t-value	p-value	OR		95%CI.low		95%CI.high
VCAM.1	0.028	0.013			2.104	0.035	1.028		1.002		1.055
ICAM.1	0.767	0.379			2.025	0.043	2.154		1.025		4.525
Ascorbate	−55.639	27.323			−2.036	0.042	<0.001		<0.001		0.0124
Outcome = ICU Care											
TSLP	0.216		0.100	2.158		0.031	1.242	1.020		1.511	
IL17c	0.131		0.067	1.959		0.050	1.140	1.000		1.300	

## 4 Discussion

In the present study we detailed the oxidative imbalance in COVID-19 patients. We demonstrate a worse clinical (30-day mortality, duration of hospitalization) and biochemical (serum inflammatory markers) phenotype in the ICU subjects compared to non-ICU patients. Transcriptionally (using mRNA sequencing), we show an enrichment of several pathways including reactive oxygen species (ROS Pathway) in ICU subjects and an enrichment of type 1 Interferon response in the non-ICU subjects. Furthermore, we demonstrate increased oxidant levels (CMH and ascorbate radicals) and decreased antioxidant levels (uric acid) in SARS-CoV-2 infected subjects compared to control subjects. Interestingly, a nonspecific measure of serum antioxidant capacity (FRAS) assay demonstrated increase levels in the ICU subjects compared to non-ICU subjects. Finally, by applying multivariate analyses we demonstrate a positive correlation of ICAM-1 and V-CAM-1 with the 30-day mortality while a negative association of ascorbate radical levels. Similarly, hospitalization was better predicted by the TSLP and IL-17c levels.

EPR-analysis clearly demonstrates that the oxidative potential of serum samples from COVID-19 patients are greater than healthy subjects. SARS-CoV-2 induced oxidant production has been reported [48]. The increased level of oxidants in the serum can potentially oxidize the proteins and other small molecules in the circulation as well as on the surface of endothelium of the vasculature. A proposed mechanism for enhanced oxidant production in COVID-19 include downregulating ACE2 after viral attachment leading to an increase in superoxide and hydrogen peroxide produced by angiotensin–II–stimulated NADPH oxidases [49,50]. Our findings of no significant difference between the levels of CMH and ascorbate radicals between ICU and non-ICU subjects is in line with a previous study finding no correlation between the disease severity and changes in redox profile in the hospitalized COVID-19 subjects [25]. These findings demonstrate an overall increased oxidant milieu which as later discussed is suitable for viral replication. The main advantage of using the EPR spin probe technique is the absence processing the serum samples, such as dilution and other modifications which could introduce artifacts to the samples. In addition, the EPR spectra are not affected by any other background signals/color. The rate constants of reaction between CMH and reactive species are high (10^3−5^ M^−1^s^−1^) which affords capacity to use much lower concentrations of the probe than conventional spin traps. The reaction between CMH and reactive oxidant species is a one electron oxidation reaction, and thus does not involve multiple reactions. And importantly, the product CM^•^ does not undergo further redox reactions to form secondary radicals. There is more than one reactive oxidant in the serum responsible for the oxidation of CMH and ascorbate, hence, the limitation of using this spin probe technique is inability to identify the specific reactive species responsible for the oxidation of CMH and ascorbate.

Nitric oxide is a well-established mediator of multiple steps in the inflammatory cascade with opposing impacts (from anti-inflammatory to pro-inflammatory) [51,52]. NO_x_ (nitrite + nitrate) measurements are commonly used as a surrogate for NO assessment due to the stable nature of reactants. We observed a significantly greater NO_x_ levels in the subjects admitted to ICU compared to non-ICU subjects. At this point it is important to note: 1) that our absolute values for NO_x_ were slightly less that previously reported as well as differences between groups are opposite of what was elegantly reported in the same report [27] which is most likely due to: 1) the absence of adding preserving agent upon sampling in our studies and 2) our samples were derived from a single point in time, admission to the hospital. Nitric oxide-induced antiviral responses through activation of iNOS are reported in multiple viral infections (e.g. HIV, vaccinia, enterovirus, corona virus) [53,54]. Interestingly, NO was shown to mediate antiviral mechanisms in case of SARS-CoV-1 infection [55,56]. These antiviral mechanisms included direct inhibition of viral replication through impacting one of the two replication related cysteine proteases encoded by viral ORF1a and by a combined action through decrease of palmitoylation levels of S protein and inhibition of membrane fusion of offspring virus S protein to host ACE2 receptor [55,56]. Indeed, NO-induced inhibition of ORF1a encoded cysteine protease in SARS-CoV-2 has been demonstrated [57,58]. Indeed, S-nitrosylation by the NO-derived metabolites has previously been proposed as a molecular bases of NO-induced antiviral responses [59]. Previous studies also demonstrated alterations in NO_x_ levels in COVID-19 subjects (either during or post infection) [27,60]. Our results agree with previously published reports of greater serum nitrate levels in critically ill subjects compared to survivors [60,61]. However, a decrease in NO levels was also reported in individuals with mild infection and was attributed to decreased endothelial NO synthase (eNOS) activity potentially due to endothelial apoptosis [27,56,62]. [55] Nevertheless, NO has been proposed as an adjuvant therapy with other antiviral agents [58,63,64]. Importantly, NO is a key player in the pathophysiology of different co-morbidities (COPD, hypertension, diabetes, COPD) that demonstrate greater sensitivity to SARS-CoV-2 infection. It is plausible that NO might play multifaceted roles ranging from early nonspecific immunity to later vasoprotective roles (regulating vascular inflammation and endothelial function) in COVID-19 subjects with or without these pathologies.

In terms of antioxidant levels, our findings demonstrate that the transcriptional upregulation/enrichment of enzymes (*CAT, GCLM, PRDX2, PRDX4, MGST1, GPX4, SOD1, PRDX1, TXN, PRDX6, SELENOS*) as well as an increased total antioxidant capacity (FRAS assay) in ICU subjects compared with non-ICU subjects. However, it is important to note that we did not detect a significant change in overall antioxidant levels in the serum in COVID-19 subjects compared with the healthy subjects. Moreover, it is important to note that uric acid levels were significantly less in COVID-19 subjects compared to healthy subjects. Uric is one of the major antioxidants in the serum [[65], [66], [67]]. These differences in results could be attributed to different readouts and sensitivity between FRAS and UA assays. Our finding of an increase in the antioxidant enzymes is potentially reactive to the increased oxidant stress as observed by the CMH and ascorbate radical detection assays. Similar increases in the antioxidant enzymes was reported previously in case of COVID-19 subjects and in case of influenza virus infection [10,16]. However, in the same study a lower plasma antioxidant capacity was reported. These differences are potentially due to different sample collection time between the two studies. Indeed, lower uric acid levels are reported in critically ill subjects and in the subjects with malaria [68,69]. Apart from its typical functions as an inflammatory mediator causing free radical production, uric is also a potent plasma antioxidant and even attributed as most important substance with antioxidant abilities in the plasma [65,67,70,71]. Interestingly, contradictory reports about the uric acid levels in the COVID-19 subjects are reported. On one hand, increased uric acid levels were associated with adverse outcomes in children hospitalized with severe COVID-19 and hyperuricemia was associated with acute kidney injury and in hospital mortality [[72], [73], [74]]. Conversely, lower uric acid levels were proposed as prognostic marker for COVID-19 and were associated with disease severity [75,76]. Lastly, our combined results may coalesce to support diminished uric acid levels seen in the ICU patients as this may be due to the elevation in circulating transition metal content. For example, uric acid is known to effectively chelate Fe (UA-Fe-UA), holding it in a less redox active state (definition of antioxidant) [77]. Our results demonstrate diminished CM^•^ and ascorbate radical intensity in ICU patients versus non-ICU and in both cases, Fe-UA interaction could be contributing to the observed diminution in radical signal. It is reasonable to assume that free uric acid may be lower in samples where there is an increase in Fe and thus an increase in UA binding; however, further work is required to define this relationship as well as the potential impact more clearly on COVID-19 pathobiology. Additional potential mechanisms to consider include UA consumption by the increased free radicals and one electron oxidation by hemoglobin and heme-peroxidases such as myeloperoxidase (MPO) [78]. Urate is a known physiological substrate for MPO and urate free radical generated by such reaction can lead to production of urate hydroperoxide which is strong oxidant and a putative intermediate in urate oxidation during inflammatory and vascular diseases [[79], [80], [81]]. Both urate and MPO are associated with adverse outcomes in cardiovascular disorders [[82], [83], [84]]. Indeed, we observed a significant upregulation of MPO in the ICU patients which further supports this potential pathway.

As oxidant imbalance and inflammatory changes go hand in hand, we observed a significant upregulation of several inflammatory cytokines that are known to impact oxidant balance in the cells. For example, IL-2 can induce NO production while IL-6 and TNF-α can stimulate superoxide production [[85], [86], [87]]. On the other hand, mitogen activated protein kinase (MAPK) activation by oxidants can lead to NF-kB activation and increase cytokine production. Indeed, both MAPK and NF-kB activation-related cytokine production has been shown in SARS-CoV-2 infected cells through down regulation of dual-specificity phosphatases (DUSPs) [88].

One of the widely studied topics has been the role of interferon signaling in the COVID-19 pathogenesis. Interferon signaling can have deleterious or advantageous impacts depending upon the stage of the infection and type of interferon produced. We demonstrate here a decrease in gene expression of Type 1 interferons (IFN-α, IFN-α2, IFN-ꞵ1 and an interferon signaling partner MAVS. These results are in line with previous studies demonstrating decreased interferon signaling in COIVD-19 patients [[89], [90], [91]]. Impaired IFN signaling was also shown to be due to either presence of autoantibodies against specific IFNs or due to genetic variants [28,[92], [93], [94], [95], [96]]. Indeed, type1 interferon autoantibodies were detected in COVID-19 subject nasal mucosa and were correlated with increased viral load [94]. It is important to note that deficiency in type1 interferon has a well-known association with enhanced susceptibility to viral infections [97]. Conversely, we found increased serum levels of IFN-γ (Type 2 Interferon) which has recently been associated with C-reactive protein response and other immune markers of poor prognosis in SARS-CoV2 infection [98]. IFN-γ is known to potentiate pro-inflammatory signaling by priming macrophages for antimicrobial actions through NO production and inhibition of NLRP3 inflammasome activation [99]. In addition, levels of several cytokines were differentially expressed between the ICU and non-ICU subjects. These cytokine data further confirmed more acute/inflammatory nature of disease in ICU subjects. Indeed, huge systemic increases in cytokine concentrations (termed as cytokine storm) has been propose as an indicator of worsened outcome after SARS-CoV-2 infection that is also indicator of development of acute respiratory distress syndrome (ARDS) and organ failure [6,100]. A significantly greater ICAM-1 levels were measured in the serum of ICU subjects and multivariate analyses confirmed it as a significant predictor of mortality. These findings agree with a previous report demonstrating its significance in predicting mortality in SARS-CoV-2 infected subjects [101].

It is important to identify some limitations of the present study. This study is based on cases that were infected with the first described strain of SARS-CoV-2 that appeared in China in December 2019. This strain generated 1–4.3% mortality rate depending on geographic locations (2, 5). Since then, many new variants have emerged with varied infectivity and disease course. Future studies should compare the oxidative imbalance in subjects infected with the other variants. Moreover, our study utilized a single sample collection approach and thus, for future studies a serial sample collection approach is recommended. All our measurements are performed on the blood samples, which has limited pathophysiological significance in the absence of tissue level readouts. Our assessment of uric acid levels were accomplished using an electrochemical detection method post HPLC separation and thus were very selective as well as sensitive; however, our reported levels of uric acid, in general, are less than those reported using data derived from clinical labs. We believe this discrepancy could be derived from the difference in methodology. For example, clinical analysis relies on the reaction of uric acid with uricase which generates hydrogen peroxide that subsequently reacts with a dye (e.g. Amplex Red) to induce a color change. As such, while appropriate for clinical SOPs, does not attain the same selectivity as the assay used herein as it can be affected by alternative sources of peroxide as well as preexisting peroxide and potential redox cycling which could all coalese to artificially elevate the indication of actual uric acid concentration. In addition, due to limitations surrounding the assessability of control subjects during COVID-19, these samples were collected before COVID-19 pandemic and thus storage may have impacted the levels of antioxidants in the serum. Although we do not anticipate this to affect results and conclusions herein, we plan to confirm/refute this potential issue in a future study whereby fresh samples are collected. Furthermore, our results indicating diminished oxidants (EPR) and diminished uric acid levels will require reexamination in greater detail to determine the relative impact of COVID-19-related hemolysis/Fe on circulating uric acid concentration in a temporal manner throughout the course of the disease process. Finally, present cohort was predominately composed of Caucasians because of their ethnic predominance in the West Virginia population. Future studies should include multicenter cohorts with significant representation of minority populations (African Americans, Hispanics, and Asians) which have been shown to have varied sensitivity to COVID-19 infection.

In summary, we demonstrate significant redox imbalance in subjects with COVID-19. Moreover, our early sampling approach helped us demonstrate a positive association between the serum levels of VCAM-1, ICAM-1 and negative association between ascorbate radical and mortality in COVID subjects. While IL-17c and TSLP levels predicted need for intensive care in COVID-19 subjects. These results imply that manipulation of oxidative stress pathways could define new therapeutic strategies to interfere with SARS-CoV-2 virus infection. However, caution should be taken to identify the exact nature of imbalance to avoid the repetition of failures using non-specific antioxidant supplementation.

## Author Contribution's

Majumder, Deepak, Aesoph, Velayutham, Ye, Mazumder, Lewis, Kodali, Roohollahi, Guo, Hu: Methodology, Investigation, Formal Analysis, Visualization, Writing original draft. Khramtsov: review and edit of first draft. Johnson: Methodology, Visualization, Writing original draft. Wen: Formal Analysis, Visualization, Writing original draft. Hadique: Conceptualization, Methodology, Investigation, Formal Analysis, Visualization, Writing original draft. Kelley: Methodology, Design, Investigation, Formal Analysis, Visualization, Writing original draft. Hussain: Conceptualization, Methodology, Investigation, Formal Analysis, Visualization, Writing original draft, Supervision, Project Administration, Acquisition of Funding. All authors read and approved the final manuscript.

## Funding statement

This study was supported by National Institute of Health funding R01 ES031253 (SH), 5U54 GM104942-05 (SH/SH, GH), P20 GM103434 (GH), R01 DK124510 (EEK), and R01 HL153532 (EEK).

## Availability of data

All sequencing data is freely available and bioinformatics code is from peer reviewed publications (referred in methods) (https://www.ncbi.nlm.nih.gov/geo/query/acc.cgi?acc=xxxxxxx). RNA-Seq sequencing data has been deposited to Gene Expression Omnibus (GEO) with accession number GSE211979.

## Declaration of competing interest

The authors declare that they have no known competing financial interests or personal relationships that could have appeared to influence the work reported in this paper.

## Data Availability

Raw and processed sequencing data is deposited in public repository GEO (GSE211979).
