# Supplementary material for: Redox imbalance in COVID-19 pathophysiology
Source: Redox Biol. 2022 Sep 11;56:102465. doi: 10.1016/j.redox.2022.102465 (PMC9464257; doi:10.1016/j.redox.2022.102465)
Supplement: Multimedia component 1 [file mmc1.pptx]

## Slide 1
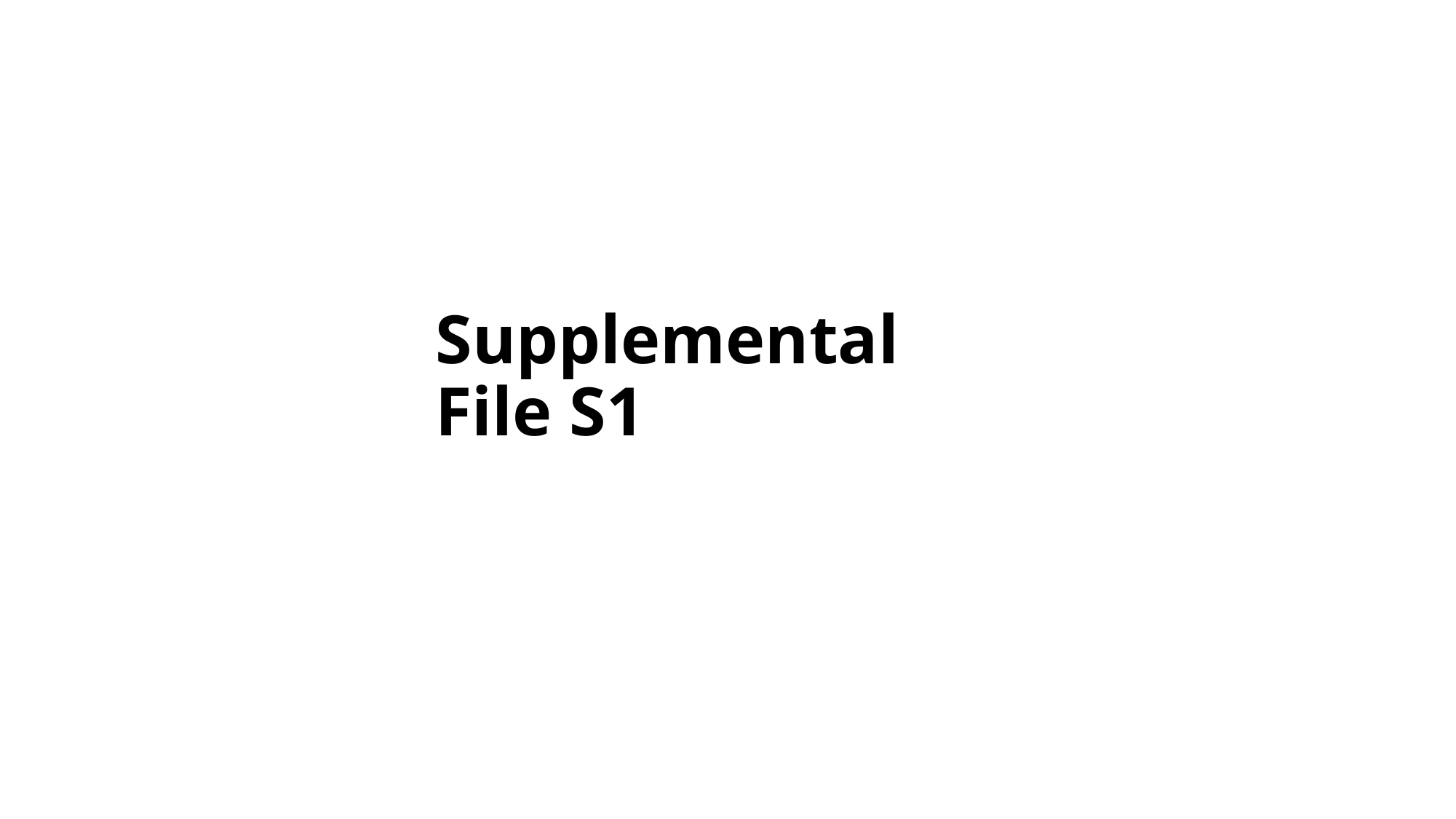

# Supplemental File S1

## Slide 2
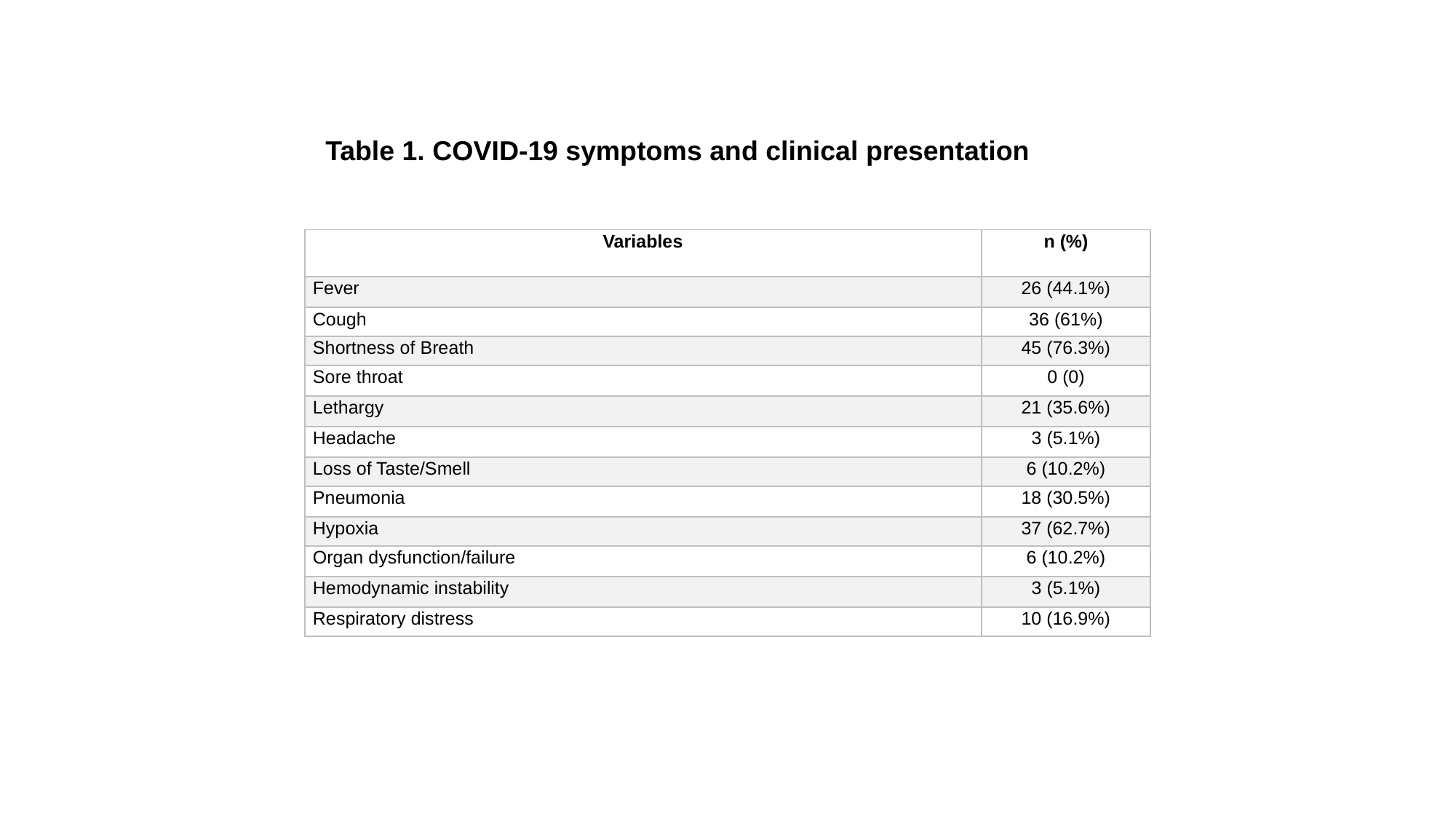

Table 1. COVID-19 symptoms and clinical presentation
| Variables | n (%) |
| --- | --- |
| Fever | 26 (44.1%) |
| Cough | 36 (61%) |
| Shortness of Breath | 45 (76.3%) |
| Sore throat | 0 (0) |
| Lethargy | 21 (35.6%) |
| Headache | 3 (5.1%) |
| Loss of Taste/Smell | 6 (10.2%) |
| Pneumonia | 18 (30.5%) |
| Hypoxia | 37 (62.7%) |
| Organ dysfunction/failure | 6 (10.2%) |
| Hemodynamic instability | 3 (5.1%) |
| Respiratory distress | 10 (16.9%) |

## Slide 3
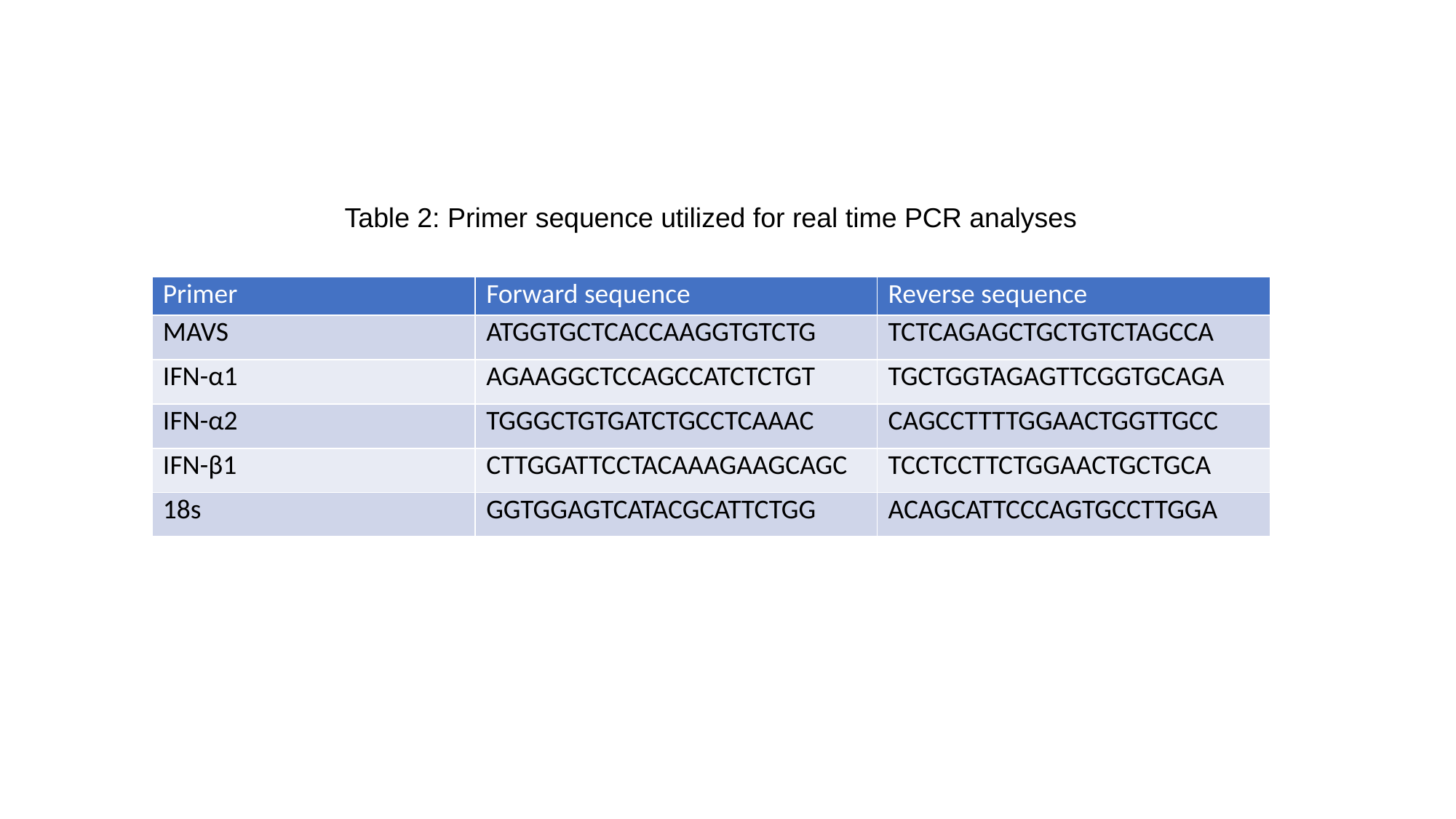

Table 2: Primer sequence utilized for real time PCR analyses
| Primer | Forward sequence | Reverse sequence |
| --- | --- | --- |
| MAVS | ATGGTGCTCACCAAGGTGTCTG | TCTCAGAGCTGCTGTCTAGCCA |
| IFN-α1 | AGAAGGCTCCAGCCATCTCTGT | TGCTGGTAGAGTTCGGTGCAGA |
| IFN-α2 | TGGGCTGTGATCTGCCTCAAAC | CAGCCTTTTGGAACTGGTTGCC |
| IFN-β1 | CTTGGATTCCTACAAAGAAGCAGC | TCCTCCTTCTGGAACTGCTGCA |
| 18s | GGTGGAGTCATACGCATTCTGG | ACAGCATTCCCAGTGCCTTGGA |
